# Impact of lengthening velocity on the generation of eccentric force by slow-twitch muscle fibers in long stretches

**DOI:** 10.1007/s00424-024-02991-4

**Published:** 2024-07-24

**Authors:** Sven Weidner, André Tomalka, Christian Rode, Tobias Siebert

**Affiliations:** 1 Department of Motion and Exercise Science, University of Stuttgart, Allmandring 28, 70569 Stuttgart, Germany; 2 Institute of Sport Science, Department of Biomechanics, University of Rostock, Rostock, Germany; 3 Stuttgart Center of Simulation Science, University of Stuttgart, Stuttgart, Germany

**Keywords:** Skeletal muscle, Contractile behavior, Stretch, Give, Soleus

## Abstract

After an initial increase, isovelocity elongation of a muscle fiber can lead to diminishing (referred to as *Give* in the literature) and subsequently increasing force. How the stretch velocity affects this behavior in slow-twitch fibers remains largely unexplored. Here, we stretched fully activated individual rat soleus muscle fibers from 0.85 to 1.3 optimal fiber length at stretch velocities of 0.01, 0.1, and 1 maximum shortening velocity, *v*_max_, and compared the results with those of rat EDL fast-twitch fibers obtained in similar experimental conditions. In soleus muscle fibers, *Give* was 7%, 18%, and 44% of maximum isometric force for 0.01, 0.1, and 1 *v*_max_, respectively. As in EDL fibers, the force increased nearly linearly in the second half of the stretch, although the number of crossbridges decreased, and its slope increased with stretch velocity. Our findings are consistent with the concept of a forceful detachment and subsequent crossbridge reattachment in the stretch’s first phase and a strong viscoelastic titin contribution to fiber force in the second phase of the stretch. Interestingly, we found interaction effects of stretch velocity and fiber type on force parameters in both stretch phases, hinting at fiber type-specific differences in crossbridge and titin contributions to eccentric force. Whether fiber type-specific combined XB and non-XB models can explain these effects or if they hint at some not fully understood properties of muscle contraction remains to be shown. These results may stimulate new optimization perspectives in sports training and provide a better understanding of structure–function relations of muscle proteins.

**Supplementary Information:**

The online version contains supplementary material available at 10.1007/s00424-024-02991-4.

## Introduction

**Fig. 1:**
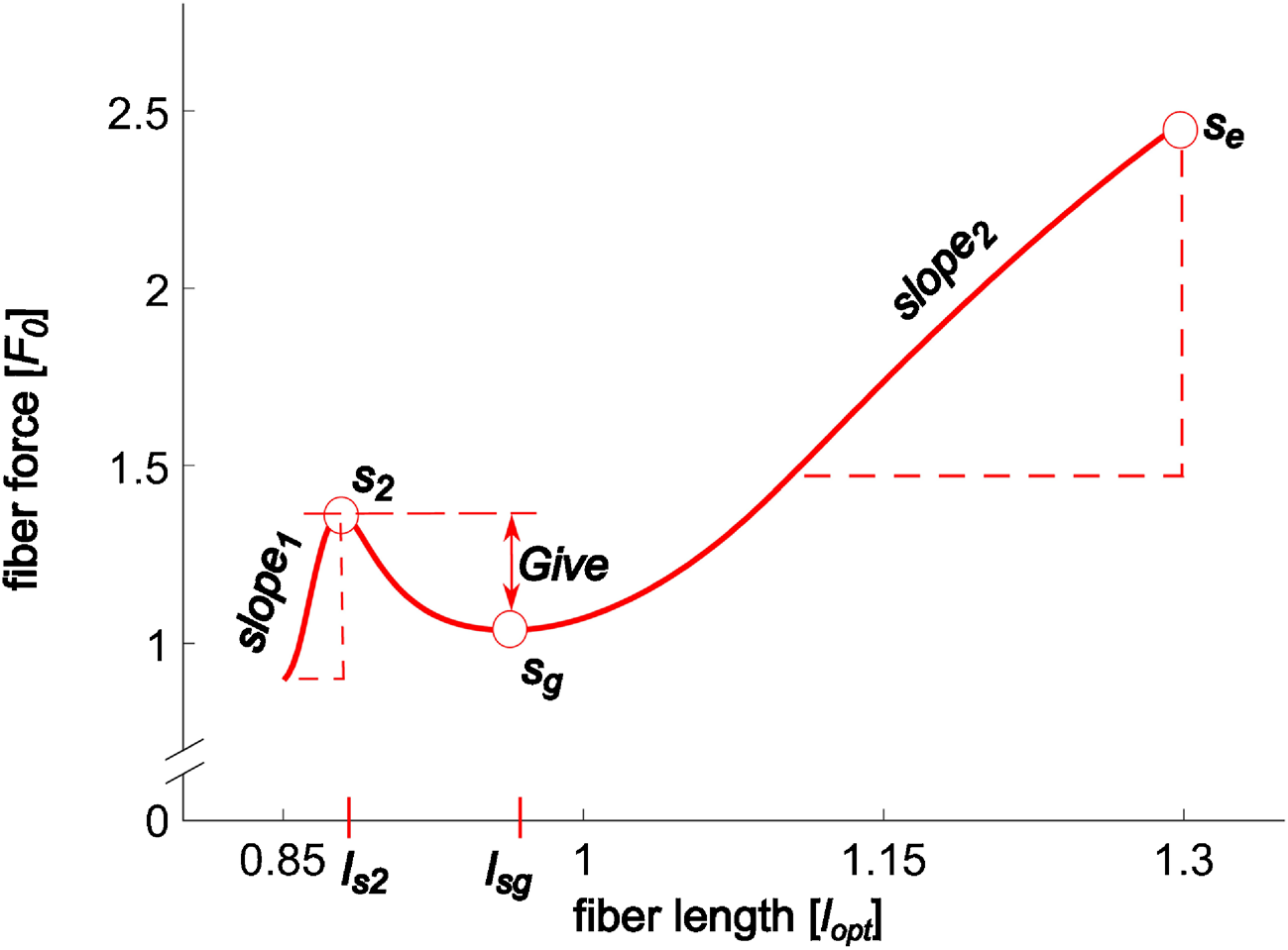
Exemplary force–length trace obtained in a long isovelocity stretch. Fiber forces are normalized to maximum isometric force, *F*_*0*_, and fiber length is normalized to optimal fiber length, *l*_opt_. Experiments start with an isometric phase until force achieves a plateau (not shown). Here, the fully activated skinned soleus fiber is then stretched from 0.85 to 1.3 *l*_opt_ at 1 maximum fiber contraction velocity. *s*_*2*_ is the first local force maximum during the stretch. *s*_*g*_ is the local force minimum during the stretch, and *Give* is the difference between *s*_*2*_ and *s*_*g*_. The force at the end of the stretch is *s*_*e*_. *slope*_*1*_ and *slope*_*2*_ are the force slopes resulting from linear regression analysis of the force–length data between the initial isometric force and *s*_*2*_ and the second half of the stretch, respectively. *l*_*s2*_ and *l*_*sg*_ are the lengths, where *s*_*2*_ and *s*_*g*_ occurred. A raw data set for three experiments with different stretch velocities is shown in Figure S1

Eccentric muscle contractions are associated with unique features compared to isometric or concentric contractions, i.e., increased force, work, and performance at decreased oxygen consumption, reduced metabolic cost (ATP), and improved energy efficiency [1, 18, 31, 43, 47, 65]. Numerous studies have shown that muscle force rises steeply during the early phase of the stretch, followed by a relatively compliant transient phase. This behavior likely depends on the stretch velocity [54, 63, 85]. More specifically, the initial steep force rise (*slope*_*1*_, Fig. 1) often ends in a characteristic force peak (*s*_*2*_), and both increase with stretch velocity [79, 85]. After this initial force increase, muscle [19, 27, 75] and fiber [10, 24, 79] forces decrease in fast stretches, called *Give* [19]. In long stretches, muscle [75] and fiber [76, 79] forces increase nearly linearly again after the *Give* phase. The corresponding force slope (*slope*_*2*_) is larger than the force slope of the underlying total (active + passive) isometric force–length relation [77]. *Slope*_*2*_ also increases with stretch velocity [79, 85]. Ample evidence supports the idea of a cumulative mechanism that combines crossbridge (XB) and non-crossbridge (non-XB) structures (e.g., titin) to the force response during active muscle lengthening [76]. While *slope*_*1*_, *s*_*2*_, and *Give* are primarily influenced by XB behavior [22, 54, 62, 79], *slope*_*2*_ depends more on non-XB structures [77, 85]. Thus, the velocity dependence of the mentioned parameters likely stems from XB and non-XB structures.

Specificities of these structures influence the velocity-dependent muscle force generation. Slow-twitch muscle fibers show lower myosin ATPase activity, lower XB-cycling frequencies, and lower maximal shortening velocities compared to fast-twitch muscle fibers [5, 8, 64]. Furthermore, different muscles differ in non-XB structures, e.g., collagenous structures (Endo-, Peri-, Epimysium [84]) or titin isoforms [23, 55, 83], resulting in different passive properties [70] impacting eccentric force generation. Recently, Weidner et al. [85] investigated the force produced by fast-twitch extensor digitorum longus (EDL) muscle fibers during long stretches over an extensive range of stretch velocities given as percentages of maximum contraction velocity, *v*_max_. *slope*_*1*_ and *slope*_*2*_ increased by 195% and 377% from 0.01 to 1 *v*_max_ stretch velocity, respectively. Furthermore, *Give*, defined as the force decrease from the initial peak to the following local minimum in force, was absent for the slow stretch velocity (0.01 *v*_max_), appeared at 0.1 *v*_max_, and increased at *v*_max_ [85]. However, it is unclear if slow-twitch muscle fibers show a similar velocity dependency of stretch-related parameters within the same experimental conditions as reported for fast-twitch fibers in Weidner et al. [85].

Thus, the study aims are (1) to examine the velocity dependence of eccentric force parameters in soleus fibers, a typical slow-twitch muscle [12], and (2) to identify fiber type-specific differences by comparison with a fast-twitch muscle. The two muscles were selected for analysis because both muscles (soleus and EDL) predominately express different MHC isoforms. The soleus muscle expresses the slow type 1 MHC isoform (96.1 ± 2.9%). In contrast, the EDL expresses predominately the fast type 2A (18.8 ± 1.7%) and type 2B (75.7 ± 2.2%) MHC isoforms [4, 71]. We performed eccentric muscle fiber stretches over the same length range (0.85 to 1.3 optimal fiber length, *l*_opt_) with equal relative stretch velocities (0.01, 0.1, and 1 *v*_max_) as Weidner et al. [85] with the fast EDL muscle fibers. While 0.01 and 0.1 *v*_max_ correspond to relative lengthening velocities respectively in slow- and fast-twitch muscles during walking [3], 1 *v*_max_ was chosen to cover sprint velocities. We normalized the stretch velocities to *v*_max_ to account for fiber type-specific differences in contraction velocity (EDL’s *v*_max_ is about fivefold higher compared to soleus). We expect fiber type-independent force characteristics in the XB-dominated short range of the stretch. Because of lower absolute velocities, we expect lower forces in slow-twitch fibers in the long range of the stretch due to the likely viscoelastic non-XB (titin) contribution to the total force [6, 11, 28, 29, 79].

## Materials and methods

### Animal and tissue preparation

Soleus muscles were extracted from seven male Wistar rats (age, 8 to 10 months; weight, 300–370 g; 12-h light to 12-h dark cycle; housing temperature, 22 °C) immediately after euthanization. The use of all animals for this study has been approved according to the German animal protection law (Tierschutzgesetz, §4 (3); Permit Number: T 201_21 ST).

The muscles were taken from the left hind limb. Permeabilized single muscle fiber preparation, storage, and activation followed [77, 78]. Fiber bundles were dissected, permeabilized in skinning solution at 4 °C for 30 min, and then stored in a 50% glycerol and 50% skinning solution mixture at − 20 °C for 6 to 8 weeks. On experiment day, single fibers were dissected from the muscle bundles with fine forceps under a dissecting microscope and cut to 1.2 mm in length. They were then clamped on both sides with aluminum foil T-shaped clips. The fibers were treated with a relaxing solution containing 1% (v/v) Triton-X 100 for 2 to 3 min at 4 °C to remove internal membranes [42].

### Experimental setup

The first step involved transferring the muscle fibers from the skinning solution to the experimental chamber of the fiber-test apparatus (600A, Aurora Scientific, Canada). Then, the fiber-clip unit was attached to a Model 403A force transducer (Aurora Scientific, Ontario, Canada) and a Model 322 C-I length controller (Aurora Scientific, Ontario, Canada). The setup was mounted on the x–y stage of an inverted Eclipse Ti-S microscope (Nikon, Japan). The fiber ends were fixed with glutaraldehyde in rigor solution, and the T-clips were secured to the apparatus with fingernail polish to enhance stability and improve mechanical performance during the experiment [30].

The sarcomere length was measured in the middle segment of the fibers. The passive fiber length was adjusted to achieve an optimal sarcomere length (${l}_{{s}_{0}}$) of 2.5 ± 0.05 µm (mean ± standard deviation) for maximal isometric force (*F*_*0*_) development [72]. The fiber width (*w*) and height (*h*) were measured at approximately 0.1-mm intervals along the entire length using a 10 × ELWD dry-objective (NA 0.60, Nikon, Japan) and a 10 × eyepiece. The fiber cross-sectional area was calculated assuming an elliptical shape (pi^.^*h*^*.*^*w*/4). A high-speed video system (Aurora Scientific 901B, Canada) combined with a 20 × ELWD dry-objective (NA 0.40, Nikon, Japan) and an accessory lens (2.5 × , Nikon, Japan) was used to visualize the striation pattern and track sarcomere length changes dynamically and accurately.

### Solutions

The relaxing solution contained (in mM) 100 TES, 7.7 MgCl_2_, 5.44 Na_2_ATP, 25 EGTA, 19.11 Na_2_CP, and 10 GLH (pCa 9.0); the pre-activating solution 100 TES, 6.93 MgCl_2_, 5.45 Na_2_ATP, 0.1 EGTA, 19.49 Na_2_CP, 10 GLH, and 24.9 HDTA; the activating solution 100 TES, 6.76 MgCl_2_, 5.46 Na_2_ATP, 19.49 Na_2_CP, 10 GLH, and 25 CaEGTA (pCa 4.5); and the skinning solution 170 potassium propionate, 2.5 MgCl_2_, 2.5 Na_2_ATP, 5 EGTA, 10 IMID, and 0.2 PMSF. The storage solution is the same as the skinning solution, except for the presence of 10 mM GLH and 50% glycerol (v/v). Cysteine and cysteine/serine protease inhibitors (trans-epoxysuccinyl-L-leucylamido-(4-guanidino) butane, E-64, 10 mM; leupeptin, 20 µg ml^−1^) were added to all solutions to preserve lattice proteins and thus sarcomere homogeneity [42, 77]. pH (adjusted with KOH) was 7.1 at 12 °C. 450 U ml^−1^ of creatine kinase (CK) was added to all solutions except skinning and storage solutions. CK was obtained from Roche (Mannheim, Germany); all other chemicals were from Sigma (St. Louis, MO, USA).

### Experimental protocol

All trials of the permeabilized fiber experiments were performed at a constant temperature of 12 ± 0.1 °C. At this temperature, the fibers were highly stable and endured active lengthening protocols and prolonged activations [56, 57]. The fibers were activated through calcium diffusion in the presence of ATP by immersing them subsequently in three different solutions: (1) a pre-activating solution for equilibration for 60 s; (2) an activation solution (pCa = 4.5) which resulted in a fast increase in force until a plateau was reached (increase in force less than 1% within 1.5 s) before stretching the fiber or keeping the fiber in isometric condition; (3) in a relaxing solution (pCa = 9.0) after the ramp or isometric experiment for 420 s. For the exact composition of the experimental solutions, see the “Solutions” section.

The experiments involved active eccentric ramps that stretched the fibers from a length of 0.85 to 1.3 *l*_opt_, a typical working range of muscles suitable for comparison with previous research [7, 85]. Single-skinned fibers were activated at 0.85 *l*_opt_ (resulting in ∼2.0-µm sarcomere length under activation) and stretched to 1.3 *l*_opt_ (resulting in ∼2.9 µm under activation) with constant stretch velocities of 0.01, 0.1, and 1 *v*_max_ (Fig. 1). *v*_max_ (0.47 ± 0.11 *l*_opt_ s^−1^; *n* = 6 fibers) of skinned soleus muscle fibers was determined in a different set of muscle fibers according to [79] and agrees with literature data [16]. After each stretch, the same ramp was performed again passively. Isometric reference contractions were performed at *l*_opt_ (2.5 ± 0.05 µm passive) before and after each ramp contraction to determine force degradation and *F*_*0*_. *F*_*0*_ was calculated as the average of isometric force before and after the stretches. Subsequently, the force responses during stretch were normalized to *F*_*0*_. In the eccentric contraction experiments, the isometric force decreased by an average of 1.55 ± 0.49% per activation (with a maximum of 10 activations per fiber). This rate of force loss is in line with other studies under similar conditions [14, 77] and demonstrates repeatable preparation routines and physiological fiber functionality. Additionally, the order of the experiments was randomized to eliminate any systematic effects of fatigue on stretch parameters. During the trials, the sarcomere length, force, temperature, and length controller position were recorded.

### Data processing and statistics

An A/D interface (604A, Aurora Scientific, Canada) recorded force and length data at 1 kHz. Real-time software (600A, Aurora Scientific) was utilized for data acquisition. MATLAB R2018a (Mathworks, Natick, MA, USA) was used to analyze the collected data through a custom-written script. The sarcomere lengths were either reported in absolute values or divided by optimal sarcomere length. Forces were divided by individual *F*_*0*_, while fiber length *l* was divided by individual *l*_opt_. The statistical analysis was carried out using SPSS 29 (IBM Corp., Armonk, NY, USA) and MATLAB R2018a (Mathworks, Natick, MA, USA).

Analogous to Weidner et al. [85], this study focused on four prominent force values (*s*_*2*_*, s*_*g*_,* Give, s*_*e*_) and two force slopes (*slope*_*1*_, *slope*_*2*_), as shown and defined in Fig. 1. We determined if these parameters, as well as the lengths *l*_*s2*_ and *l*_*sg*_ (where *s*_*2*_ and *s*_*g*_ occurred), varied with stretch velocity. One-way repeated measures ANOVAs explored the effect of *stretch velocity* on these parameters. In case of significant differences, Tukey’s HSD was used for post hoc analyses. Effect sizes for one-way ANOVA were classified as small (*η*^*2*^ < 0.06), medium (0.06 ≤ *η*^*2*^ ≤ 0.14), and large (*η*^*2*^ > 0.14), based on Cohen’s classification [13].

Two-way mixed ANOVAs explored the combined effects of *stretch velocity* and *fiber type* (our slow-twitch [soleus] fibers vs previously published fast-twitch [extensor digitorum longus, EDL] fibers [85]) on the force values and force slopes. In the case of a significant interaction or significant main effects, we report significant simple main effects of *stretch velocity* for each fiber type (one-way repeated measures ANOVA) and of *fiber type* for each stretch velocity (independent *t*-tests). Interactions are divided into the following categories according to [40]: Ordinal interactions are defined by a consistent rank order of treatment levels across all factor levels, with lines in interaction plots never crossing, and main effects are generally consistent and interpretable. Disordinal interactions exhibit a change in rank order, visualized by crossing lines on the plots, indicating non-monotonic relationships between factors, and main effects may be overshadowed by the interaction. Hybrid interactions combine elements of both, with rank order consistency in some factors and variability in others, resulting in mixed plots where lines may cross in some graphs but not in others, and main effects are partially interpretable depending on the factor investigated. To account for multiple comparisons, we used post hoc analyses. Results were expressed as mean ± standard deviation.

## Results

**Fig. 2:**
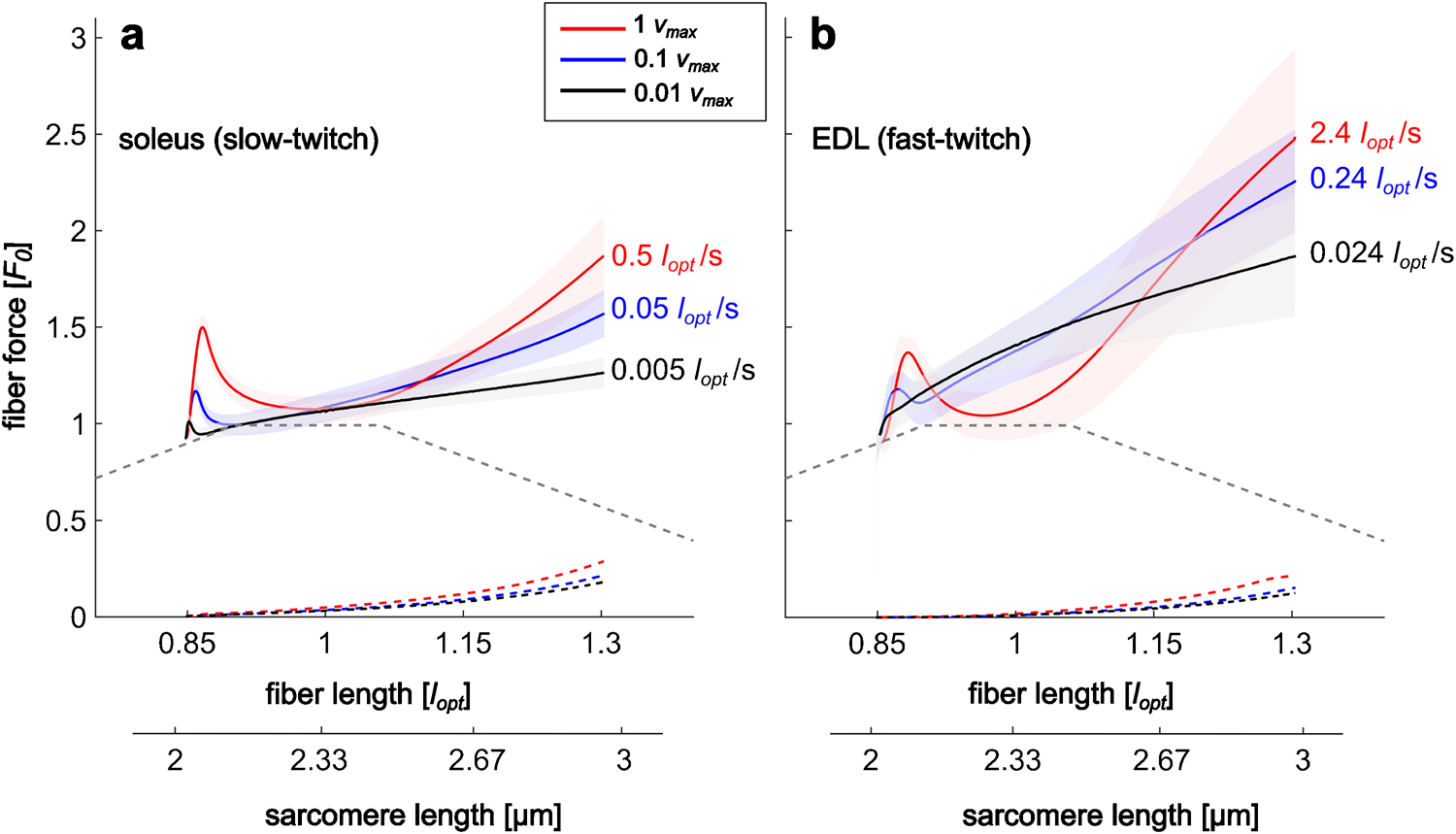
Force–length traces of fully activated slow- and fast-twitch fibers during long isovelocity stretches. Total fiber forces (active + passive force), *F*, are normalized to maximum isometric fiber force, *F*_*0*_; fiber lengths, *l*, are normalized to optimal fiber length, *l*_opt_. Ensemble averages (solid lines) and variances (shadowed areas) of active stretch forces and corresponding ensemble averages of passive stretch forces (dashed lines) are shown for stretch velocities of 0.01 (black), 0.1 (blue), and 1 *v*_max_ (red). Soleus data (slow-twitch, **a**) from this study and EDL data (fast-twitch, **b**) from [85] were obtained under similar experimental conditions. For orientation, figures include a schematic active isometric force–length relationship (gray dashed line). An enlarged section of the region of *slope*_*1*_ can be found in Figure S2

The soleus fibers (*n* = 27) generated a maximum isometric tension of 98.6 ± 18.6 kN m^−2^ at *l*_opt_ and a maximum contraction speed, *v*_max_, of 0.47 *l*_opt_ s^−1^ (EDL [85]: 2.42 *l*_opt_ s^−1^). The fiber cross-sectional area was 0.006 ± 0.002 mm^2^. The total force during isovelocity stretches initially increased steeply and linearly; then, the force declined and increased again, showing a local minimum (Fig. 2a). All observed parameters (cf. Figure 1) increased significantly with stretch velocity (Table 1; Figure S3, S4, S5). The main effect of *stretch velocity* and subgroup comparisons were all significant with *p* < 0.001.

**Table 1:** Statistical analysis of the effect of *stretch velocity* on slow-twitch soleus fiber force parameters in isovelocity stretches

*v*_max_		Mean (± standard deviation)			*p-*value|*F-*value, *η*^*2*^	Relative change (%)		
		0.01	0.1	1		0.01 to 0.1	0.1 to 1	0.01 to 1
*slope*_*1*_	*F*_*0*_*/l*_*s0*_	9.34 (± 1.45)	16.57 (± 1.58)^1^	31.39 (± 3.44)^1,2^	< 0.001|837, 0.97	77	89	236
*s*_*2*_	*F*_*0*_	1.01 (± 0.03)	1.17 (± 0.04)^1^	1.51 (± 0.06)^1,2^	< 0.001|2136, 0.988	16	29	50
*l*_*s2*_	*l*_opt_	0.85 (± 0.00)	0.86 (± 0.00)^1^	0.87 (± 0.00)^1,2^	< 0.001|2398, 0.989	1	1	2
*s*_*g*_	*F*_*0*_	0.94 (± 0.03)	0.99 (± 0.05)^1^	1.07 (± 0.07)^1,2^	< 0.001|89, 0.774	5	8	14
*l*_*sg*_	*l*_opt_	0.87 (± 0.00)	0.9 (± 0.01)^1^	0.99 (± 0.02)^1,2^	< 0.001|922, 0.973	3	10	14
*Give*	*F*_*0*_	0.07 (± 0.02)	0.18 (± 0.04)^1^	0.44 (± 0.06)^1,2^	< 0.001|1323, 0.981	157	144	529
*slope*_*2*_	*F*_*0*_*/l*_*s0*_	0.34 (± 0.09)	0.93 (± 0.18)^1^	1.89 (± 0.36)^1,2^	< 0.001|695, 0.963	174	103	456
*s*_*e*_	*F*_*0*_	1.27 (± 0.08)	1.57 (± 0.12)^1^	1.87 (± 0.19)^1,2^	< 0.001|393, 0.938	24	19	47

All one-way repeated measures ANOVAs and post hoc comparisons of subgroups were highly significant (*p* < 0.001) and effects were strong (*η*^*2*^ > *0.14*). ^1^A significant difference compared to 0.01 *v*_max_; ^2^a significant difference compared to 0.1 *v*_max_*.* Maximum isometric force, *F*_*0*_; optimal sarcomere length, *l*_*s0*_; maximum contraction speed, *v*_max_. The last three columns show relative changes in the dependent variable (left column) values from 0.01 to 0.1, 0.1 to 1, and 0.01 to 1 *v*_max_. For dependent variable definitions, see Fig. 1

**Fig. 3:**
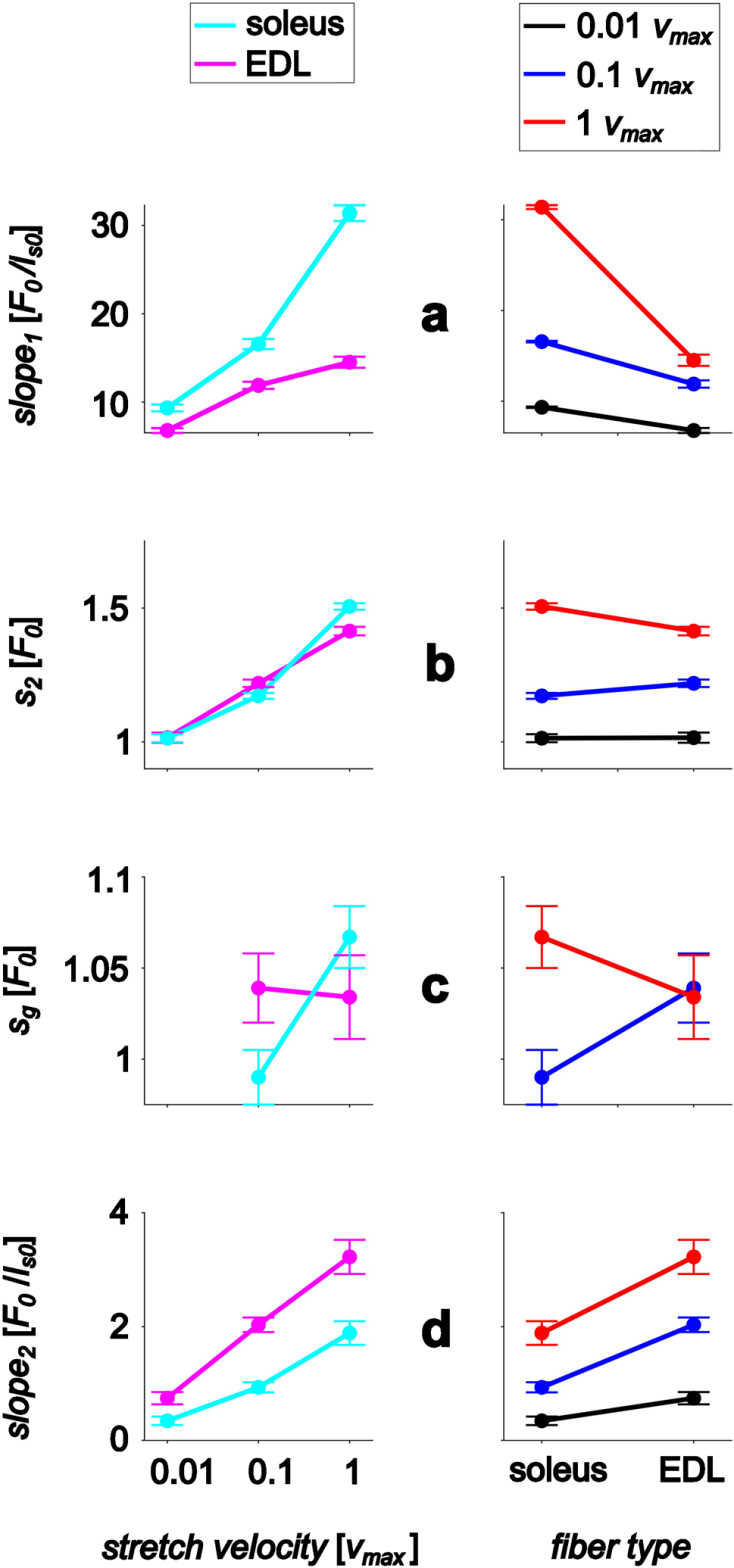
Interaction *stretch velocity* × *fiber type* on force parameters. Subgroup means (points) and 95% confidence intervals (error bars) are shown. *Stretch velocity* and *fiber type* exhibit ordinal interaction on the initial force rise, *slope*_*1*_ (**a**); the force slope in the last half of the stretch, *slope*_*2*_ (**d**); hybrid interaction (only stretch velocity is interpretable) on the peak force *s*_*2*_ (**b**); and disordinal interaction (no main effect is interpretable) on the local force minimum *s*_*g*_ (**c**). *Stretch velocity* has a main effect on *slope*_*1*_, *s*_*2*_, and *slope*_*2*_ (a, b, d right). *fiber type* has a main effect on *slope*_*1*_ and *slope*_*2*_ (a, d left). Maximum isometric fiber force, *F*_*0*_

The slow-twitch (soleus) fiber force traces in this study showed similar qualitative behavior as the fast-twitch (EDL) fibers from a previous study [85] (Fig. 2). The force–length traces of single EDL and soleus muscle fibers during stretches with constant velocity did not reflect the changes in the slope of the underlying active isometric force–length relationship (Fig. 2b). This behavior was consistent across all stretch velocities tested (0.01, 0.1, and 1 *v*_max_). Instead, force increased steeply and linearly, with a temporary decline observed for the two higher velocities. Beyond a local minimum (*s*_*g*_), force increased until the end of the stretch, exceeding initial forces by 80% (for 0.01 *v*_max_) to 150% (for 1 *v*_max_). No *s*_*g*_ was observed for the lowest stretch velocity (0.01 *v*_max_). However, unlike EDL, soleus fibers showed a force peak *s*_*2*_ and *Give* at 0.01 *v*_max_ stretch velocity. Table 2 summarizes the results of our statistical comparison of soleus and EDL parameters. *Stretch velocity* and *fiber type* interacted with four (*slope*_*1*_, *slope*_*2*_, *s*_*2*_, and *s*_*g*_) out of six variables (Fig. 3).

**Table 2:** Statistical comparison of slow-twitch soleus fibers and fast-twitch EDL fiber data

	*Fiber type*	*Stretch velocity*			*p*-value|*F*-value		
					*η*^*2*^		
		0.01 *v*_max_	0.1 *v*_max_	1 *v*_max_	*Fiber type*	*Stretch velocity*	Interaction
*slope*_*1*_ (*F*_*0*_*/l*_*s0*_)	Soleus	9.34 (± 1.45)	16.57 (± 1.58)^1^	31.39 (± 3.44)^1,2^	< 0.001|211	< 0.001|457	** < 0.001**|668
	EDL	**7.62 (± 1.97)**	**9.69 (± 3.03)**^**1**^	**14.05 (± 2.62)**^**1,2**^	0.847	0.923	0.762
*s*_*2*_ (*F*_*0*_)	Soleus	1.01 (± 0.03)	1.17 (± 0.04)^1^	1.51 (± 0.06)^1,2^	0.33|0.96	< 0.001|643	** < 0.001**|16.2
	EDL	1.06 (± 0.11)	**1.22 (± 0.08)**^**1**^	**1.41 (± 0.07)**^**1,2**^	0.023	0.940	0.283
*s*_*g*_ (*F*_*0*_)	Soleus	0.94 (± 0.03)	0.99 (± 0.05)^1^	1.07 (± 0.07)^1,2^	0.74|0.12	0.008|7.76	**0.003**|9.74
	EDL	-	**1.08 (± 0.12)**	1.03 (± 0.12)	0.144	0.159	0.192
*s*_*e*_ (*F*_*0*_)	Soleus	1.27 (± 0.08)	1.57 (± 0.12)^1^	1.87 (± 0.19)^1,2^	< 0.001|89.0	< 0.001|108	0.45|0.75
	EDL	**1.87 (± 0.31)**	**2.22 (± 0.29)**^1^	**2.49 (± 0.47)**^1,2^	0.685	0.724	0.018
*Give* (*F*_*0*_)	Soleus	0.07 (± 0.02)	0.18 (± 0.04)^1^	0.44 (± 0.06)^1,2^	< 0.001|22.8	< 0.001|451	0.223|1.53
	EDL	-	**0.14 (± 0.09)**	**0.38 (± 0.10)**^b^	0.358	0.917	0.036
*slope*_*2*_ (*F*_*0*_*/l*_*s0*_)	Soleus	0.34 (± 0.09)	0.93 (± 0.18)^1^	1.89 (± 0.36)^1,2^	< 0.001|151	< 0.001|325	** < 0.001**|19.1
	EDL	**0.72 (± 0.40)**	**2.03 (± 0.46)**^**1**^	**3.15 (± 0.76)**^**1,2**^	0.800	0.895	0.335

The last three columns show the *p*-values, *F*-values, and effect sizes (ES, partial eta-squared) of the two-way mixed ANOVAs pertaining to the main and interaction effects of *fiber type* and *stretch velocity*. ^1^A significant difference compared to 0.01 *v*_max_; ^2^a significant difference compared to 0.1 *v*_max_; values in bold, sig. different from soleus (*p* < 0.05) and sig. interactions. Maximum isometric force, *F*_*0*_. For dependent variable (left column) definitions, see Fig. 1

Table 2 indicates that both the EDL and soleus exhibit similar behavior concerning the parameters *slope*_*1*_, *s*_*2*_, *s*_*e*_, and *slope*_*2*_. Both muscles demonstrate a significant increase in these parameters with increasing stretch velocity. However, for *s*_*g*_ and *Give*, there is no consistent behavior. The EDL only exhibits *Give* at 0.1 *v*_max_ and 1 *v*_max_, while the soleus shows *Give* at all three tested velocities. Additionally, there are no differences in the level of *s*_*g*_ for the EDL. In contrast, the soleus shows a significant increase in *s*_*g*_ with increasing stretch velocity.

The significant interaction effects for *slope*_*1*_, *s*_*2*_, *s*_*g*_, and *slope*_*2*_ shown in Table 2 are visually depicted in Fig. 3. The force slopes exhibit an increase with stretch velocity. While *slope*_*1*_ is higher for the soleus, *slope*_*2*_ is higher for the EDL. For *s*_*g*_, the main effects cannot be interpreted due to the intersection in both graphs. Finally, *s*_*2*_ depends solely on the velocity of stretching and not on the fiber type.

## Discussion

This research on slow-twitch muscle fibers of the rat soleus extends a recent study [85] that investigated the total force response of fast-twitch rat muscle fibers (EDL) under similar experimental conditions. Our investigations reveal three major similarities of the force produced by fully activated slow-twitch fibers compared with fast-twitch fibers [77, 85] during long isovelocity stretches (Fig. 2): *(i)* the forces first increase, then fall (except for EDL fibers at 0.01 *v*_max_) and rise before or within the force–length relationship’s (FLR’s) plateau region depending on stretch velocity and increase in the range of the FLR’s descending limb; *(ii)* the force slopes in the range of the FLR’s descending limb increase with stretch velocity; *(iii)* all tested force and length parameters increase with stretch velocity (except for *s*_*g*_ in EDL fibers).

Moreover, some differences between fiber types in short-range parameters persisted despite normalized contraction velocities that aimed at accounting for differences in absolute *v*_*max*_.

### Impact of stretch velocity in slow-twitch fiber experiments

Interestingly, for all stretch velocities tested, the force–length traces (colored lines) did not reflect slope changes of the underlying FLR and increased in the range of the FLR’s descending limb (Fig. 2a). In addition, force slopes in the range of the FLR’s descending limb increased with stretch velocity. These findings are in contrast with classic theories of muscle contraction [33–35] that would, e.g., predict a slope change in force when the number of XBs decreases (during the transition from the FLR’s plateau to its descending limb) and force slopes that decrease with stretch velocity in the range of the FLR’s descending limb. Commonly applied Hill-type muscle models [25, 67, 69, 81] approximating classic theories of muscle contraction represent neither the decrease of force in the FLR plateau during the stretch nor force slopes increasing with stretch velocity in the range of the FLR’s descending limb. This especially hampers simulations of movements involving fast muscle stretches induced by perturbations during locomotion [2] or large muscle stretches, e.g., accident predictions of multi-body models (e.g., OpenSim: [67], Anybody: [15, 58]).

#### XB contribution to eccentric force generation

We found significant increases in the initial force increase’s slope (*slope*_*1*_), its maximum (s_*2*_*),* and the length where this maximum occurred (*l*_*s2*_*)* with increased stretch velocity (Table 1, Fig. 3). Assume that the S2 region of the myosin molecule exhibits purely linear elastic behavior, and that the rate of detachment is constant for eccentric contraction as in Huxley’s classical crossbridge (XB) model (Huxley, 1957). Pulling a single XB with increasing speed, the detachment force *s*_*2*_ as well as *l*_*s2*_ would increase. However, different from our results, *slope*_*1*_ would remain constant. Stretching a cohort of XBs with increasing speed as we did when stretching fibers, a smaller fraction of XBs would be detached at a given length because less time passed when reaching this length, resulting in more XBs contributing in parallel to force generation. Thus, *slope*_*1*_ would increase in faster stretches. However, when comparing the slope within the first 20% of the *slope*_*1*_ period, there is no significant difference between the tested velocities (*F*(1.47,38.28) = 0.465, *p* = 0.57). This behavior suggests that a similar number of XBs are stretched for all three velocities during this period. Further experiments (with higher sample rates and more tested velocities) and modeling are required to explain the finer details of our results, like differences and similarities between fiber types.

Similar behavior has also been found in other studies [22, 54, 85] and has been explained with the viscoelasticity of XBs [9] or a non-XB component [49]. The observed length change associated with the development of *s*_*2*_ was less than 0.05 *l*_*opt*_, which is consistent with previous studies that stretched intact and skinned muscle fibers from various species [22, 45, 73, 79]. It is assumed that *l*_*s2*_ relates to the forcible detachment of XBs bound to actin [19, 45]. We would argue that it is within the capacity of the Huxley XB model to explain the increase of *slope*_*1*_*, s*_2_*,* and *l*_*s2*_ when considering the dynamic equilibration of the XB distribution from the isometric to the eccentric condition.

As in our study (Table 1, Figure S5c), long stretches of muscle fibers resulted in *Give* across different species and stretch velocities [19, 36, 38, 45, 85]. The detachment of XBs followed by a time-dependent restoration of a steady-state XB distribution may account for increased *l*_*sg*_ with stretch velocity.

#### Non-XB contribution to eccentric force generation

Non-XB structures such as titin [37, 82] might complement the force recovery and dominate the force at longer elongation. Besides its fundamental role in organizing and maintaining sarcomeres, titin performs intricate and diverse functions in muscle contraction [21, 51, 76]. Titin interacts with many muscle proteins [44] and aids in generating force when the muscle is actively stretched. Titin’s force generation mainly relies on titin-actin binding during Ca^2+^ activation [6, 11, 17, 28, 46]. Several studies [75, 77, 85] report a quasilinear increase in force during the second half of a long stretch (*slope*_*2*_). Since XBs would contribute forces that decrease with contraction speed in the range of the FLR’s descending limb, the observed increase in *slope*_*2*_ (Table 1, Figure S3b) and the force *s*_*e*_ at the end of the stretch (Table 1, Figure S4c) are consistent with a strong, linear viscoelastic titin contribution to fiber force [11, 29].

### Comparison between soleus (slow-twitch) and EDL (fast-twitch) muscle fiber kinetics during stretch

By normalizing to *v*_max_ (resulting in fivefold absolute stretch velocities for fast-twitch EDL fibers compared with slow-twitch soleus fibers), we aimed to balance the effects of fiber type. Though we found qualitatively similar behavior of slow- and fast-twitch fibers (Fig. 2), our experiments revealed some differences between the two fiber types (Table 2). The slow-twitch soleus force–length traces show comparably low inter-subject variance. This holds even when considering absolute stretch speeds. For example, absolute stretch speed 1 *v*_max_ in soleus is about twofold that of 0.1 *v*_max_ in EDL; regardless, the variance in the red data in Fig. 2a (soleus, 1 *v*_max_) is lower than in the blue data in Fig. 2b (EDL, 0.1 *v*_max_). The high variance in fast-twitch fiber forces has been documented in both individual muscle fibers and fiber bundles [55]. This points to a higher variability in passive structures and titin isoforms in fast-twitch muscles, especially because the contribution of non-XB structures to eccentric muscle force [77] and the variance (Fig. 2) increase with muscle length [59]. However, it should be noted that the EDL expresses two myosin isoforms [4, 71], which may contribute to the observed variability.

XB kinetics probably dominate the steep initial force increase and the force peak *s*_*2*_ and partially the local force minimum *s*_*g*_ (and hence *Give*). This is supported by recent studies who investigated stretches in fiber bundles and single fibers with and without XB inhibitors [29, 63, 77]. Their results showed that, in the presence of crossbridge inhibitors, there was no steep initial increase in force, followed by a subsequent decrease. Accordingly, *slope*_*1*_, *s*_*2*_, and *Give* increase with stretch velocity (Table 2) for both fiber types. However, the factors *fiber type* and *stretch velocity* revealed ordinal interaction on *slope*_*1*_ and hybrid interaction on *s*_*2*_. *slope*_*1*_ increased stronger with stretch velocity for the soleus (Table 2, Fig. 3a) compared to EDL. Further, soleus’ *s*_*2*_, while similar for 0.01 v_max_, was significantly lower for 0.1 *v*_max_ and higher than EDLs’ *s*_*2*_ for 1 v_max_ (Fig. 3b). Hence, normalization to *v*_max_ did not alleviate fiber type-related XB effects. Moreover, *fiber type* and *stretch velocity* revealed disordinal interaction on the local force minimum during the stretch, *s*_*g*_ (Fig. 3c). This might be due to two nonlinear processes, the restoration of a steady-state XB distribution and viscoelastic non-XB dynamics, which contribute to force redevelopment. It remains to be shown whether such interactions can be explained by a fiber model accounting for XB and non-XB dynamics or if they hint at unknown features of muscle contraction.

Interestingly, forces do not drop significantly below the maximum isometric force (cf. *s*_*g*_ in Table 2) at any stretching rate in both fiber types (Fig. 2). Possibly, titin-actin interactions [60] secure a certain force level to prevent damage. During activation, titin has been suggested to bind to actin [17, 39, 53, 74], thereby reducing the free titin spring length and increasing titin force in subsequent stretches [52, 60, 66]. The resulting titin force during fiber stretch could protect the muscle function when XBs tear off. With the attachment of new XB and the further increase in activation-dependent titin forces (that overcompensate the loss due to decreasing numbers of XB in the FLR’s descending limb range), the muscle can generate large forces and thus effectively avoid overstretching.

In this context, it is known that changes in titin-based stiffness are likely to play an important role in adjusting the passive and active properties of skeletal muscle in health and disease (for a detailed review on posttranslational modifications of titin, see [21]). It is also known that slow-twitch muscles (e.g., soleus [80]) usually express long titin isoforms accounting for low titin-based passive tension. In contrast, fast-twitch muscles such as the rabbit psoas muscle [20, 32, 55, 83] predominantly express shorter titin accounting for higher passive forces [50]. This is in line with higher values of *slope*_*2*_ in EDL than soleus fibers in quasistatic stretches at 0.01 *v*_max_ (Fig. 3d). However, further studies are needed to test the idea that the interaction of XB and non-XB structures represents a protective muscle adaptation to prevent destruction.

In the second half of the stretch, titin becomes even more important for force generation [77]. Soleus and EDL show increasing *slope*_*2*_ and force at the end of the stretch, *s*_*e*_, with increasing relative stretch velocity (Table 2, Fig. 2). Hence, the viscoelastic non-XB contribution to fiber force outweighs the force slope-decreasing effect of XBs in the range where XB numbers decline [33–35] (the FLR’s descending limb). As expected due to the viscoelastic behavior of non-XBs, fivefold higher absolute stretch velocity leads to a larger *slope*_*2*_ and *s*_*e*_ in the EDL compared with the soleus stretch experiments (Table 2). It is also noteworthy that the interaction between crossbridges and non-crossbridge components (e.g., titin) leads to an approximately linear increase in force during the second half of the stretch (Fig. 2). However, all titin segments show a nonlinear (exponential) increase in force when stretched in isolation [41]. Further development of muscle models that take activation-dependent titin-actin interactions into account [26, 48, 52, 60, 66] is necessary. In particular, a fiber type-specific adaptation of these models could contribute to better predictions of musculoskeletal models [61, 68]. Incorporating an activation-dependent titin spring into a muscle model [60] already leads to linear titin force during stretch. How this translates to varying stretch speeds and the observed viscoelastic nature of non-XB contributions is not yet clear.

## Conclusions

Both slow-twitch and fast-twitch fiber forces in long isovelocity stretches at different velocities are qualitatively similar. They increase sharply, decline (*Give*) except for EDL at 0.01 *v*_max_, and recover with a positive slope in the length range where XB numbers decrease. Consistent differences in force parameters between fiber types and some interaction effects of *stretch velocity* and *fiber type* on these parameters highlight differences between slow- and fast-twitch fibers in the XB-dominated short range and the non-XB-dominated long range of the stretch. Whether a fiber type-specific combined XB and non-XB model can explain these effects or if they hint at some not fully understood properties of muscle contraction remains to be shown. Moreover, despite the well-established occurrence of *Give* in situ and in vitro, *Give* is yet to be established in vivo to understand its significance for regular muscle function.

## Supplementary Information

Below is the link to the electronic supplementary material.

Supplementary file Plot of an exemplary raw data set. The upper graph shows the force-time data over an entire activation of all three stretch velocities (red: 1 vmax, blue: 0.1 vmax, black: 0.01 vmax). The middle graph shows the sarcomere length-time graph of the experiment. The lower graph shows the relative length-time data corresponding to the upper and middle graphs 1 (JPG 1454 KB)

Supplementary file Plot of enlarged first part of the stretch. The figure shows an enlargement of the initial stretch phase. To illustrate slope1, linear regressions between the start of the stretch and s2 were drawn (dashed lines), which are used to calculate slope1 according to [7] 2 (JPG 157 KB)

Supplementary file The force slopes increased with stretch velocity. Force slope units are maximum isometric force, F0, per optimal sarcomere length, ls0; stretch velocities are normalized to maximum contraction velocity, vmax. stretch velocity had a significant effect (*p* < 0.001) on the initial force slope (slope1, a) and that during the second half of the stretch (slope2, b).*** indicate significant (*p* < 0.001) differences between subgroups. For statistical details see Table 1 3 (JPG 1194 KB)

Supplementary file The force maxima increased with stretch velocities. Forces are normalized to maximum isometric force, F0, stretch velocities to maximum contraction velocity, vmax. stretch velocity had a significant effect (*p* < 0.001) on the force peak s2 (a), the corresponding sarcomere length ls2 (b), and the force at the end of the stretch, se (c). *** indicate significant (*p* < 0.001) differences between subgroups. For statistical details see Table 1 4 (JPG 1025 KB)

Supplementary file The local force minimum and Give increased with stretch velocity. Forces are normalized to maximum isometric force, F0, stretch velocities to maximum contraction velocity, vmax. stretch velocity had a significant effect (*p* < 0.001) on the local force minimum sg (a), the corresponding sarcomere length lsg (b), and the force decrease Give (c). *** indicate significant (*p* < 0.001) differences between subgroups. For statistical details see Table 1 5 (JPG 700 KB)

## Data Availability

The data presented in this study are available on request from the corresponding author.
