# Supplementary figures and images for: Impact of lengthening velocity on the generation of eccentric force by slow-twitch muscle fibers in long stretches
Source: Pflugers Arch. 2024 Jul 24;476(10):1517–27. doi: 10.1007/s00424-024-02991-4 (PMC11381483; doi:10.1007/s00424-024-02991-4)

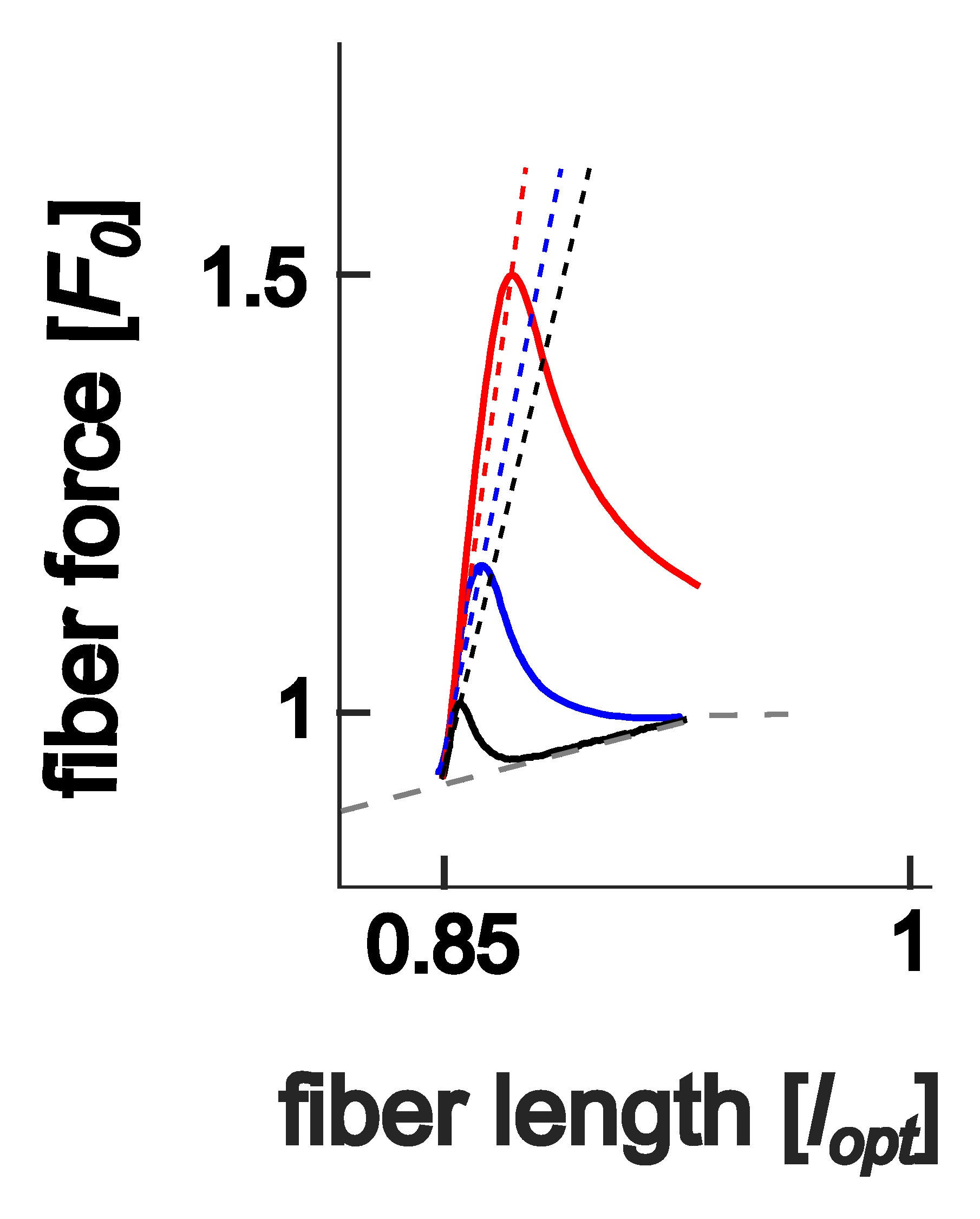

Supplement: Supplementary file 2 — Supplementary file Plot of enlarged first part of the stretch. The figure shows an enlargement of the initial stretch phase. To illustrate slope1, linear regressions between the start of the stretch and s2 were drawn (dashed lines), which are used to calculate slope1 according to [7] 2 (JPG 157 KB) [file 424_2024_2991_MOESM2_ESM.jpg]

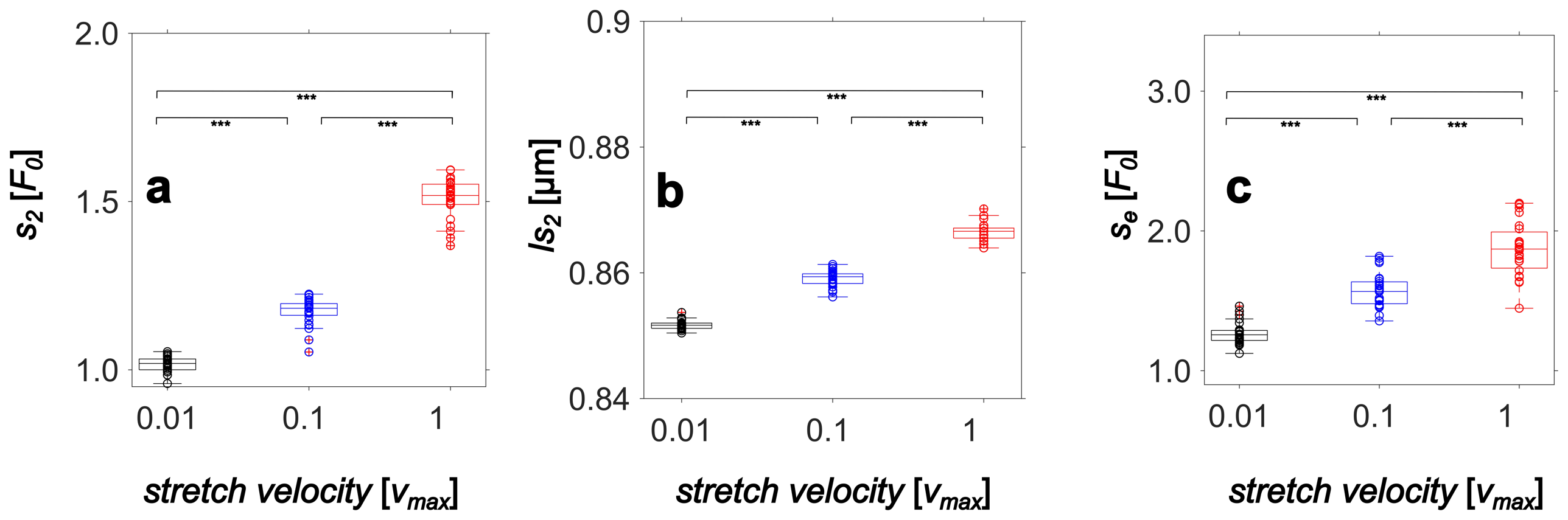

Supplement: Supplementary file 4 — Supplementary file The force maxima increased with stretch velocities. Forces are normalized to maximum isometric force, F0, stretch velocities to maximum contraction velocity, vmax. stretch velocity had a significant effect (p < 0.001) on the force peak s2 (a), the corresponding sarcomere length ls2 (b), and the force at the end of the stretch, se (c). *** indicate significant (p < 0.001) differences between subgroups. For statistical details see Table 1 4 (JPG 1025 KB) [file 424_2024_2991_MOESM4_ESM.jpg]

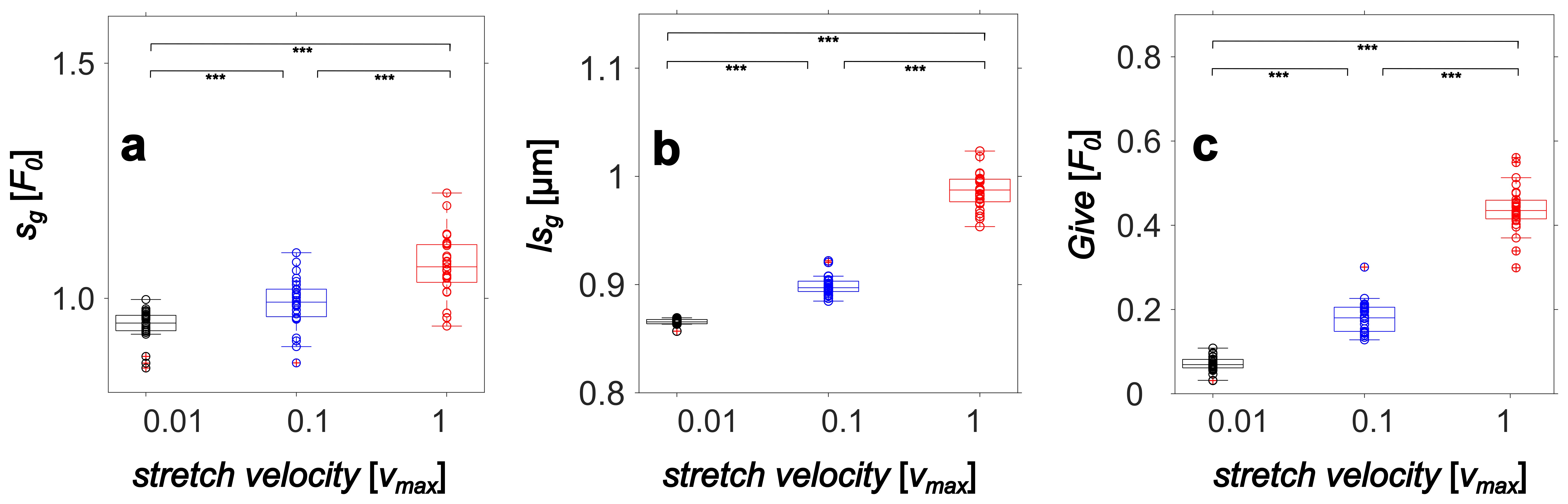

Supplement: Supplementary file 5 — Supplementary file The local force minimum and Give increased with stretch velocity. Forces are normalized to maximum isometric force, F0, stretch velocities to maximum contraction velocity, vmax. stretch velocity had a significant effect (p < 0.001) on the local force minimum sg (a), the corresponding sarcomere length lsg (b), and the force decrease Give (c). *** indicate significant (p < 0.001) differences between subgroups. For statistical details see Table 1 5 (JPG 700 KB) [file 424_2024_2991_MOESM5_ESM.jpg]
